# Hematidrosis as a manifestation of COVID‐19 containment‐induced stress

**DOI:** 10.1002/jha2.45

**Published:** 2020-11-06

**Authors:** Christian Récher

**Affiliations:** ^1^ Service d'Hématologie Centre Hospitalier Universitaire de Toulouse Institut Universitaire du Cancer de Toulouse Oncopole Université Toulouse III Paul Sabatier Toulouse Toulouse France


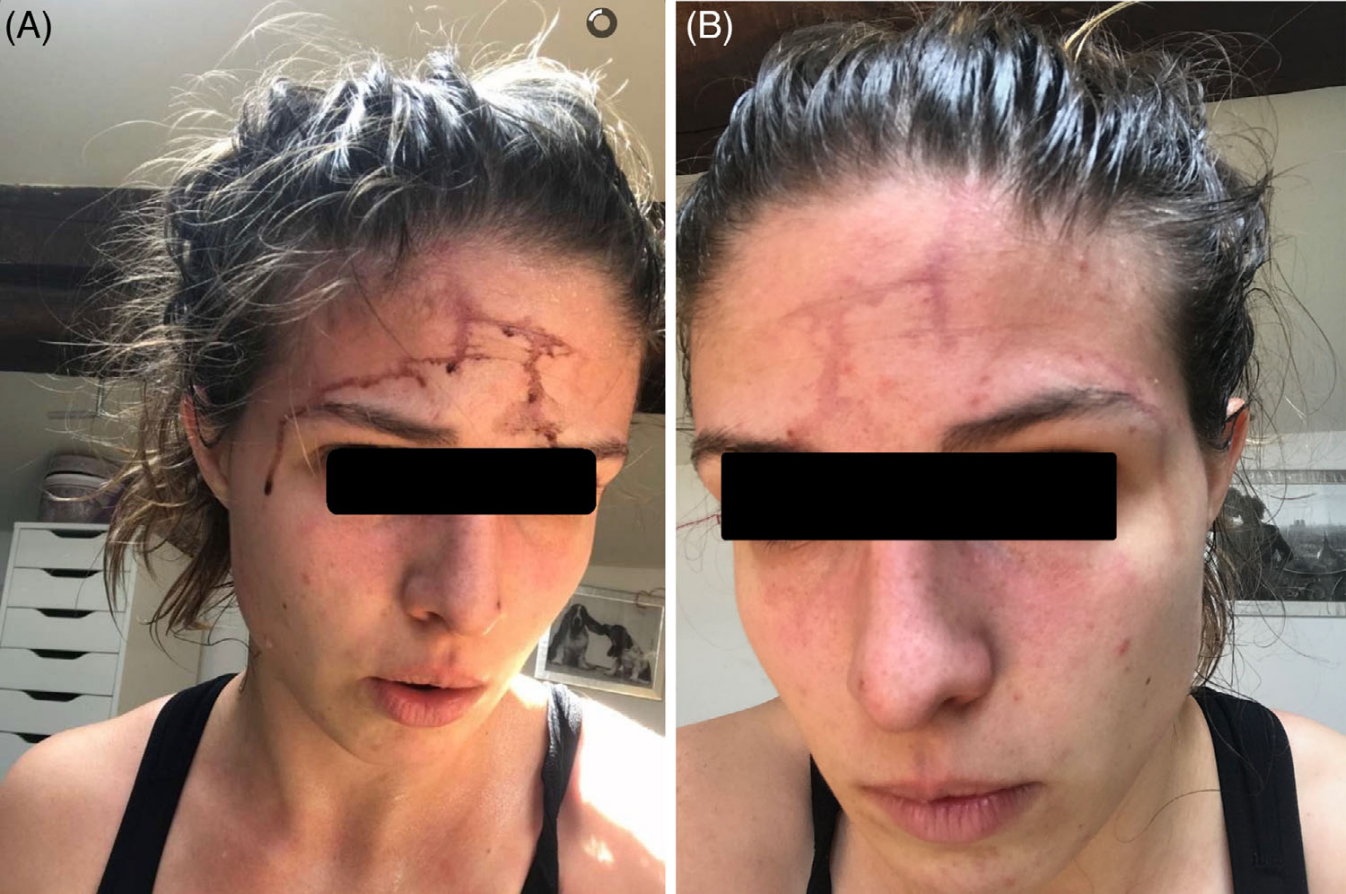


A 22‐year‐old woman presented with bloody sweats during intense physical exertion (A). After wiping her forehead, a vascular pathway remained apparent for 24 hours (B). This was her first episode of hematidrosis. Physical examination was normal. Platelet count, hemostasis parameters, iron status, biochemistry, and protein electrophoresis were normal. No treatment was done. She reported considerable stress during the COVID‐19 pandemic containment period due to conflicting family relationships.

Hematidrosis is an eccrine sweat disorder characterized by isolated or recurrent episodes of spontaneous bloody sweating. This very rare phenomenon has been mainly described in young women facing a stressful situation. Hematidrosis has not been associated with an underlying somatic disease. The physiopathology is unknown. Management with psychotherapy, beta blockers, or anxiolytics has been proposed in cases with recurrent episodes.

